# Support for children identified with acute flaccid paralysis under the global polio eradication programme in Uttar Pradesh, India: a qualitative study

**DOI:** 10.1186/1471-2458-12-229

**Published:** 2012-03-22

**Authors:** Rie R Yotsu, Katharine Abba, Helen Smith, Abhijit Das

**Affiliations:** 1 Department of Dermatology, National Center for Global Health and Medicine, Shinjuku-ku, Tokyo 162-8655, Japan; 2 International Health Group, Liverpool School of Tropical Medicine, Pembroke Place, Liverpool L3 5QA, UK; 3 Centre for Health and Social Justice, Avenue 21, G Block, Saket, New Delhi 110017, India

**Keywords:** Acute flaccid paralysis, AFP Surveillance, Healthcare, India, Poliomyelitis, Pulse Polio Programme, Residual paralysis

## Abstract

**Background:**

Cases of polio in India declined after the implementation of the polio eradication programme especially in these recent years. The programme includes surveillance of acute flaccid paralysis (AFP) to detect and diagnose cases of polio at early stage. Under this surveillance, over 40,000 cases of AFP are reported annually since 2007 regardless of the number of actual polio cases. Yet, not much is known about these children. We conducted a qualitative research to explore care and support for children with AFP after their diagnosis.

**Methods:**

The research was conducted in a district of western Uttar Pradesh classified as high-risk area for polio. In-depth interviews with parents of children with polio (17), with non-polio AFP (9), healthcare providers (40), and key informants from community including international and government officers, religious leaders, community leaders, journalists, and academics (21) were performed.

**Results:**

Minimal medicine and attention were provided at government hospitals. Therefore, most parents preferred private-practice doctors for their children with AFP. Many were visited at homes to have stool samples collected by authorities. Some were visited repetitively following the sample collection, but had difficulty in understanding the reasons for these visits that pertained no treatment. Financial burden was a common concern among all families. Many parents expressed resentment for their children's disease, notably have been affected despite receiving multiple doses of polio vaccine. Both parents and healthcare providers lacked information and knowledge, furthermore poverty minimised the access to available healthcare services. Medicines, education, and transportation means were identified as foremost needs for children with AFP and residual paralysis.

**Conclusions:**

Despite the high number of children diagnosed with AFP as part of the global polio eradication programme, we found they were not provided with sufficient medical support following their diagnosis. Improvement in the quality and sufficiency of the healthcare system together with integration of AFP surveillance with other services in these underprivileged areas may serve as a key solution.

## Background

Poliomyelitis, or polio, is a viral infectious disease in children transmitted faecal-orally. It causes acute flaccid paralysis (AFP), a clinical manifestation characterized by weakness of the lower extremities with reduced muscle tone, which sometimes leads to lifelong residual paralysis or death. Prevention by vaccines is essential, as there is no curative treatment. Supportive treatments are the only treatment available for polio, including analgesics, physiotherapy, and good nutrition for better immunity [1,2].

In 1988, the global polio eradication programme was launched to eradicate polio. The programme strategy includes: 1) immunisation of children aged < 1 year with oral polio vaccine (OPV), 2) National Immunisation Days targeting children aged < 5 years with OPV, 3) surveillance of outbreaks, and 4) immunisation with 'mop-up' doses of vaccine at time of outbreaks [3]. Despite these intensive attempts to eradicate polio, wild poliovirus is still endemic in India together with Afghanistan, Nigeria, and Pakistan [4]. The Pulse Polio Immunisation Programme started in India in 1995 with the objective of 100% coverage of OPV [5]. Reported cases successfully declined from over 500 cases annually to 42 in 2010 [6]. In 2011, there was only one case reported, bringing the country close to a milestone for eradication of polio - a 12-month period with zero case of polio [7]. Regardless of this success, the eradication programme needs to remain in place to provide response to re-outbreaks as well as mop-up immunisation, and surveillance of new cases and monitoring in collaboration with the National Polio Surveillance Project.

Active surveillance for polio searches for AFP, an early sign of infection by poliovirus. When a child with AFP is identified, the case is reported to the local government hospital. Then, two stool samples are collected either at hospital or at their homes and sent to a designated laboratory for diagnosis. Number of cases registered as AFP is showing significant increase from 8,500 cases in 2003 to over 40,000 cases per year starting from 2007 onwards, regardless of the number of actual confirmed polio cases [6]. This rise in AFP cases is attributed to significant improvements in the quality of the surveillance system [8]. Notwithstanding this large population of children diagnosed with AFP, very little is known about their post-diagnosis outcomes.

In this study, we aimed to explore what kind of support children with AFP were able to receive after their diagnosis under the global polio eradication programme through their experiences, their families' and healthcare providers' views on the given situation, and suggestions for change.

## Methods

### Study sites

The study was conducted in Uttar Pradesh in North West India from April to June 2008. The state has the largest population in the country accounting for 16.4% of the country's 1.1 billion population, with high population density and low sanitation [9,10]. Per capita income of Uttar Pradesh was 9,753 rupees (US$ 231.93), 45.7% lower than the national level, and 32.8% of its population were estimated to be living below poverty line in 2005 [9]. Most cases of polio in India are reported either from Uttar Pradesh or Bihar, and these two places are known to be the last pockets for wild poliovirus [4,6]. In our study, we purposively selected the district of Muzaffarnagar as representative of the whole Uttar Pradesh, in terms of socio-economic development, geographic, and transportation condition [9,11]. It is classified as a high-risk area for polio, and new polio cases followed almost the same trend as those in other high-risk areas [12].

### In-depth interviews

We obtained patient information for cases of AFP for the past three years (2005-2007) through primary health centres (PHCs) and local NGOs. We were able to identify 21 out of the 82 confirmed polio cases (25.6%) during this period. In-depth interviews were carried out with 17 sets of parents; four declined to participate. We also interviewed nine sets of parents of children with AFP, but whose stool exams were negative for wild poliovirus (non-polio AFP). To capture a range of experiences and views of the healthcare providers', including community health workers and other prominent community members, we recruited various key informants based on their experience/insight in relation to their work, social position, and proximity to community members: 26 frontline healthcare providers (*i.e*., auxiliary nurse midwives (ANMs), accredited social health activists (ASHAs), and Aganwadi workers who were the local female caregivers), 8 primary health centre (PHC) doctors, 4 private-practice doctors, 2 traditional healers, 4 international organisation members, 2 government officers, 3 religious leaders, 4 community leaders, 2 journalists, and 6 academics. All key informants we approached agreed to be interviewed.

Standard topic guides were used to ensure relevant areas to be covered during interviews, including patients' and healthcare providers' experiences and views about: healthcare provided for children with AFP, their difficulties and needs, patient management, and views about the quality of healthcare system (See Additional file 1: Table S1: Topic guidelines for interviews with the parents with AFP; and Additional file 1: Table S2: Topic guidelines for interviews with healthcare workers and key informants). Interviews with parents were conducted in Hindi, lasted between 20-40 minutes, and were held in participants' homes or otherwise in a quiet place in their neighbourhood. For key informants, a total of 30 interviews were conducted in English and the rest in Hindi, lasted between 30-60 minutes, and were held in health centres or in their offices. All participants gave informed consent for voice-recording their interviews. Recruitment and interviews were conducted until the study reached its saturation point. Two bilingual (Hindi/English speaking) researchers trained and experienced in qualitative research conducted all the interviews.

### Analysis

The Framework approach was applied to analyse the qualitative data obtained from in-depth interviews [13], and MAXqda software (VERBI GmbH, Marburg, Germany) was used for coding, retrieving data, and merging of the researchers' analysis. Voice-recordings in Hindi were translated and transcribed verbatim into English by a professional translator. Five interviews in Hindi (3 interviews with parents and 2 interviews with key informants) were randomly selected and were independently translated by the bilingual researchers to check for consistency with the work of the translator. English recordings were transcribed by the researchers.

Through the process of transcribing, reading, and rereading of transcripts, emerging key ideas, concepts, and themes were identified and made into a list which the team reached consensus after a number of discussions; this formed the basis of the thematic framework. Transcriptions were then imported into MAXqda and were coded using a coding system developed according to the thematic framework (36 codes for parents; 47 codes for key informants). One researcher was responsible for coding all the interviews.

The frequency of the coded segments and relations between the segments were examined, and a paper chart was used to rearrange and combine the codes. Through this process, six main themes were identified. A matrix was constructed for each theme, followed by a list of sub-issues and pertinent data from the interviews. This allowed all researchers to check for consistency, similarity, and diversity within and across groups and themes that we all agreed upon. Thus, ensuring validity and trustworthiness of our data.

### Ethics

The research protocol was approved by the ethics committee of the Liverpool School of Tropical Medicine, UK, and by the Centre for Health and Social Justice, India.

## Results

Among the 17 cases of confirmed polio, 11 were male and six were female (Table 1). Five cases had residual paralysis. Among the children with non-polio AFP, seven cases were male and two were female (Table 2). Two children had residual paralysis. The backgrounds of families for both groups were similar; most children came from poor families where their parents worked as labourers and were illiterate. All children received multiple doses of polio vaccine, but for the three cases that lacked data from their medical records (See Additional file 1: Table S3: Expanded data for characteristics of children in the study; and Additional file 1: Figure S1: Map of Muzaffarnagar and cases of polio). The six main themes identified in the analysis are presented below together with illustrative quotes. We maintained the wordings in the quotes as close to the local dialect of Hindi spoken in the study area.

**Table 1:** Characteristics of confirmed polio cases in the study

**Case No**.																	
	
	1	2	3	4	5	6	7	8	9	10	11	12	13	14	15	16	17
Gender	F	F	M	F	M	F	M	M	M	F	M	M	M	M	M	F	M
Age at interview	1.5 yr.	3.5 yr.	3 yr.	2 yr.	2 yr.	4 yr.	2 yr.	2.5 yr.	2 yr.	6.5 yr.	2 yr.	2.5 yr.	2 yr.	2.5 yr.	2.5 yr.	3 yr.	9 yr.
Age at onset	1 yr.	2.5 yr.	2 yr.	11 m	1 yr.	2.5 yr.	1.5 yr	1.3 yr.	1.5 yr.	6 yr.	10 m	1.5 yr.	6 m	1.8 yr.	1.7 yr.	2 yr.	8 yr.
Residual paralysis	No	No	No	No	No	No	No	Yes	Yes	No	No	Yes	Yes	No	No	Yes	No
Type of poliovirus*	P3	P3	P3	P3	P3	P3	P3	P1	P1	P3	P3	P1	P1	P1	P3	P1	P3
OPV doses before illness*	8	14	13	7	6	15	13	10	8-10	NA	NA	6-8	16+	9	14	14	15
Religion	Muslim	Muslim	Muslim	Muslim	Muslim	Muslim	Muslim	Muslim	Muslim	Muslim	Muslim	Hindu	Hindu	Muslim	Muslim	Muslim	Muslim
Occupation of parents	labourer	labourer	juice vendor	labourer	labourer	labourer	labourer	labourer	labourer	labourer	labourer	labourer	labourer	shop owner	labourer	labourer	rickshaw puller
Literacy of parents	illiterate	illiterate	literate	literate	literate	illiterate	illiterate	illiterate	illiterate	illiterate	illiterate	illiterate	literate	literate	illiterate	literate	illiterate

* Data retrieved from medical records of each patient

** NA: not available

**Table 2:** Characteristics of non-polio AFP cases in the study

Case Number									
	
	1	2	3	4	5	6	7	8	9
Gender	F	M	M	M	M	F	M	M	M
Age at interview	2 yr.	1.5 yr.	1.9 yr.	4 yr.	8 m	4.5 yr.	3 yr	5 yr.	6 yr.
Age at onset	1.5 yr.	1.9 yr.	6.3 yr.	3.5 yr.	5 m	4 yr.	3 yr.	4.3 yr.	6 yr.
Residual paralysis	No	Yes	No	No	No	No	No	Yes	No
OPV doses received before	12	15	25	21	NA	20	15	25	20
Religion	Muslim	Muslim	Muslim	Muslim	Muslim	Muslim	Hindu	Hindu	Hindu
Occupational of parents	Furniture shop	tailor	driver	labourer	labourer	labourer	Sugar mill employee	labourer	labourer
Literacy of parents	literate	illiterate	illiterate	literate	illiterate	illiterate	literate	illiterate	illiterate

* Data retrieved from medical records of each patient

** NA: not available

### Limitations at government hospitals

Many parents preferred to visit private-practice doctors (clinics in villages/towns), where they initially visited when they noticed their children's illness. However, for there was a high level of awareness among all kinds of healthcare workers that every suspicious case of polio must be sent to government hospitals, most families had experience of visiting government hospitals (PHCs, Community Health Centres, or District Hospital). Nonetheless, majority of the families reported medical care provided there was inadequate; hence, there was 'no peace of mind' at government hospitals. They claimed that no medicine was given, and many had a feeling that no attention was paid to their children. Some were told that their children could not be treated there because it was 'beyond their capacity'. There seemed to be little interaction between doctors and families. Most families mentioned they were only given their children's diagnosis by checking the stool, and no other services or support. This being the case, many families went back and continued visiting private-practice doctors for treatment of their paralyzed children.

"The government doctors refused. They didn't give any medicine. They didn't give injection nor did they give any tonic for strength. They said that his prevention is the only cure for this. They just did stool checking...Hospital provided no help."

- Father of 4 year-old child with polio No.6

Q) "What was your experience at the government hospital?"

A) "Once I took, and I never took him to that hospital again. If I am taking him to that hospital and getting no medicine and have to buy from outside and he is not getting any relief, it is better I go to the private hospital."

- Mother of 2.5 year-old child with polio No.8

"I saw her leg, it kind of limped aside. Then I called her father and sent them to government hospital. She went there and doctors made her walk; she could walk slowly. They gave medicine for one day and she came back. We went there again next day and then they refused. They said she could not be treated here, it was beyond their capacity. You may go and show her someplace else."

- Mother of 2 year-old child with polio No. 4

"After waiting for a long time, it's just they take your hands and give medicine. There is no interest. It's not okay, it's a polio patient so examine her carefully or give attention. That's not there."

- Father of 3 year-old child with polio AFP No.16

"They said it's your child and you can show him where ever you like. He doesn't have dangerous polio*. It's still in limits and the child will be cured. He will be all right. After that they didn't give any medicine or anything...They said you can show him where ever you like. And there is nothing specific. He may or may not be cured."

*The child was affected with wild type 3 poliovirus. Type 3 is considered to be less severe compared to Type 1 with less incidence of paralytic poliomyelitis. Approximately 80% of cases infected with type 1 cause paralysis, while it is 13% for type 3 [14].

- Mother of 2 year-old child with polio No. 11

Q) "Why you didn't continue going to the government hospital?"

A) "We didn't get relief, nor did we feel comfortable. Then we decided to go to private. They didn't value us in the government hospital. They said 'I have given medicine, take them back home,' whether my child is feeling good or not."

- Father of 4 year-old child with non-polio AFP No.4

In contrast, whilst government healthcare providers articulated the importance of AFP surveillance system for eradicating polio, the majority also believed the system was functioning to the benefit of children with AFP. They noted these children received special benefits like 'diagnosis free-of-cost' and 'attention of doctors', which one would not have received if they did not fall under the system. They also mentioned about prescribing medicines free-of-cost, but the reality was that they were often out of stock and had to ask the parents to purchase them outside at nearby pharmacies.

"Government doctors diagnose and they send the children back home, that's all. In most of the patients, which we have been referring to hospitals, they will take samples of stools, they will diagnose whether it is a case of polio or not, and they ask them to go back to their doctor. Most of the time, they are not giving any treatment to that patient, or patient may not be following that may be the reason. But most of the time, I have seen that patients come back to us only without any treatment."

- Private doctor No.2

"As you know, AFP cases are very important for us. We are giving more and more and more attention to that child, so I think there is no lacking or there is no gap for the health point of view for that child."

- PHC doctor No.5

"Staff at PHCs says Pradhans-ji (community leader) what can we do, we are getting only this quantity and quality of medicine from the above."

- Community leader No.4

"You see, people who go there [PHCs] are mostly poor people and our supply is also not that good; and moreover we have limited stock. They demand for good medicine, but what we have is the Government's supply only."

- International organization No.2

### Home visits by government healthcare providers

Most families recalled frequent visits made by government healthcare providers, *i.e*., doctors and ANMs, after their children were diagnosed with AFP. Doctors not only from their nearby PHCs, but also from district hospitals and sometimes from outside the district came and surprised the families. Many families did not understand why they were visited because no medicines, prescriptions, or medical support was provided for their children. Except for the first few visits to collect stool samples, the subsequent visits were typically described by the families as 'doctors come, just glance at the child, and go'. Some received the result of stool exam on paper, but meant nothing to them due to their illiteracy. There were few cases where they were given medicines or prescriptions, which they were grateful for. No family complained of discomfort at being visited by healthcare providers.

"Teams of doctors came many times in cars, sometimes four sometimes two, sometimes six for checking. They checked. They all came to see our child. But nothing at all was given. They would come and see, check his leg, take measurement and all and go back."

- Father of 1.5 year-old child with polio No.1

"We took her many times to government hospital, but they didn't give medicine. They didn't even speak there. They kept coming here [house] and took information as to how is she and all...But nobody gave medicine, nor nothing else."

- Mother of 6.5 year-old child with polio No.10

"One lady doctor had come from the city, but we didn't understand anything that day. They didn't give any medicine nor did they.....We didn't understand anything what they were saying."

- Grandmother of 4.5 year-old child with non-polio AFP No.6

Q) "Did the government doctor come to visit you?"

A)" Yes. They have visited many times, but they haven't for one month. Many big big doctors have come to see my child. One of the big doctors gave a bottle of medicine and my child got much relief with that medicine."

- Mother of 2 year-old child with polio No.13

Q) "When you gave for the stool test, during that duration, did anybody visit you?"

A) "Yes, a doctor from Charthawal had come."

Q) "What did he say?"

A) "He said the child is fine, and he gave me a prescription."

- Grandmother of 3 year-old child with non-polio AFP No.7

On the other hand, several families reported being visited only once or twice by healthcare providers after their child was diagnosed with AFP, solely for collecting stool samples.

Q) "After the child was diagnosed and stool sample was sent to Mumbai till final report came, did anybody come?"

A) "Nobody came in between that period to enquire about our child. They have said that we will take care, don't take the child anywhere, but nobody came. They came after 2.5 months later to only give report. Nobody came after collection of sample."

- Mother of 8 month-old child with non-polio AFP No.5

"No, no doctor came........No, one had come only once. After that, nobody came."

- Mother of 3.5 year-old child with polio No.2

Government healthcare providers stated that they usually followed up children diagnosed with AFP by visiting their houses. Still, some notes found in the medical records stating the absentee of the child at time of visit, *e.g*. 'child not found at this address twice', and the fact that many families were labourers, moving from one area to another, indicated the difficulty in following up the children despite their effort at times.

Q: "Do you do follow up?"

A:" Yes."

Q: "How do you follow up? What all do you do in follow up?"

A: "First, we get the stool sample with 24 hours interval. Today we gave him vaccine carrier which is specifically for stool in which we have given four solid ice packs. And we have given stool kit. Next, our worker will go and change the ice pack and will explain then how stool sample has to be collected. When our stool collection is over then we do follow up for two months or 60 days so that we can confirm whether he has residual paralysis or not."

Q: "During this period you ask patient to come to you or you go and visit him?"

A: "Mostly we go to their house. In 60 days whenever our routine immunization or other program happens, we go and see him and after 60 days we anyway go to see."

- PHC doctor No.4

### Financial burden of caring for children with AFP

We found most children affected with AFP came from lower to middle class families with poor living conditions, and most families mentioned about the financial burden of caring for children with AFP. Costs were incurred by families because they preferred going to private-practice clinics. Some families were using government hospitals, but they still had to pay for transportation and medicines, which was a burden for such families. Some families were living in remote places, and had to pay for accommodation fees along with transportation. Several families changed doctors many times to seek for treatment for their children, seeing their children not recovering; due to the nature of the disease itself.

Q) "You didn't get any support?"

A) "I did it all on my own, even though I don't have enough money to manage it."

- Father of 8 month-old child with non-polio AFP No.5

Q) "Now where the child is getting treatment?"

A) "My child is getting treatment from Muzaffarnagar and Saharanpur and various other places."

Q) "Treatment was done in government or in private clinics?"

A) "It was all private. We are poor people, we laboured a lot and spent all that money. But no relief has happened to the child."

- Mother of 3.5 year-old child with polio No.2

Q) "Then where did you take her?"

*A) "We went to one doctor nearby 'Chudiwala (bangle seller)', a local doctor. Then we went to a place called 'Sona Majha'; its medicine for polio is popular"*.

Q) "What did doctor say?"

A) "Doctor gave medicine and injections and said that she will be all right. We went there 2-3 times."

Q) "Was there any improvement?"

A) "Very little. From there we also took her to the hospital in Haridwar. They took X-ray and they tied brick from here and hung the leg."

- Father of 9 year-old child with polio No.17

Most families earned only 50 rupees (US$ 1.19) to 100 rupees per day from labour work, while being charged several times more than their daily living cost per visit to doctors. There was no financial support provided to any of the families interviewed in this study. Sometimes families had to take loans, unwillingly discontinue treatment for their children, or limited treatment for their children according to their budget.

"We were provided with no, no help at all. Our daily income is 50-100 rupees and so either we spend on food or on doctors. A lot of money was spent on this child..."

- Grandmother of 2 year-old child with non-polio AFP No.7

"We stopped since we can't afford it anymore. Either we can eat or give her medicine. We have not been able to pay off money we had taken earlier for her treatment. Now you see what happens in 50 rupees earning."

- Grandmother of 2 year-old child with polio No.7

Q) "So, where are you taking your child for treatment?"

Mother) "I'm taking my child to a private doctor."

Father) "I don't stay here at home. I am doing labour work regarding wood, so she has managed it by taking debt."

- Parents of 5 year-old child with non-polio AFP No.8

### Challenges for prevention and treatment of polio

One of the major concerns among both the families and the healthcare providers regarding the polio-affected children was the fact that despite their history of receiving over 10 to 20 doses of polio vaccines, they were still affected. Simultaneously, there was grief and anger among the families, having their promise broken that polio vaccine, or 'polio drops' as locally called, would save their children from getting paralyzed. However, many families were not able to obtain satisfying answers when they asked why their children were affected to healthcare providers. At times, no answer was given, and sometimes the blame was put on the families. The latter included possibility of mothers missing out polio drops - which they were sure they had not-- and possibility of their children not having been swallowing polio drops properly. Nonetheless, the most common answer was that it was in 'God's hands' or it was their 'destiny'.

"I asked them how can she have polio. She has been taking drops and she had vaccinations. Her name was written down when she was born. When she was born lady doctor (ANM) had come to house and gave her drops and also wrote her name. So I asked then how can she have polio, you tell me? If we are not giving drops and child gets affected we can't do anything. They said it's type 3. I don't know what that is. We are illiterate people. We don't know anything."

- Grandmother of 2 year-old child with polio No.7

"I then asked [the doctor] when I have never stopped him from taking polio drops, then how come he got polio! We gave injection and medicine (polio drops), then tell me how he got polio. Now treat him, give him medication for his disease.""[The government doctor said] what you have to do with that. Now, what has happened has happened. You might be in contact with some polio people in your neighbourhood and your child might have got germ from there. Now tell me how you can stop children. Who gets what from whom, how would you know? Who can stop them? They are children, you see, and they go out of house, no matter how much you try to stop them."

- Mother of 3 year-old child with polio No.3

"They said: 'what can we do?' The doctors here said its god's will what we can do. They said there will be no complaints from this side as all your children had taken medicine (polio drops)."

- Father of 2.5 year-old child with polio No.15

"They just said it was his destiny. Now we can't do anything and you can't do anything. They would just say that and leave."

- Mother of 2 year-old child with polio No.13

A challenge was shared among all healthcare providers that there was no definite treatment for polio, and this was all they could give as an answer when they were questioned by the families about the disease and about the multiple doses of polio vaccines. Likewise, some healthcare providers were questioning and were puzzled themselves about the need for multiple doses of polio vaccine and its ineffectiveness at times.

"Parents just want their child to become normal. They ask if they should give some kind of oil massage and so forth, but we tell them no that they should not do anything and there is no treatment for this."

- ANM No.4

"We explain to parents that for this problem we will have to do stool test and after that only we can confirm whether this is due to polio or whether there is some other problem. If it is polio, we try to explain them that there is no definite treatment. But if deformity is too much and calipers are required, well that is to be seen later. Still we are facing problem with those vaccines which need multiple doses, like 10 times, more than 10 times, not a single administration, but we can't give an explanation for this."

- PHC doctor No.1

### Awareness for available healthcare and support

Experienced elderly doctors and those called the 'polio experts' by the community people were able to give proper information and treatment for polio and other causative diseases for AFP. However, they all shared a challenge of difficulty in explaining about the disease and its treatment to the illiterate parents, and also to make the treatment affordable for the poor patients. Several healthcare providers pointed out that many of the children diagnosed with AFP were malnourished, and hence stressed the importance of nutrition. A few parents mentioned that their child improved from medications given by the doctor, and had shown the medication or the prescription during the time of interview. Following quote by one private-practice paediatrician with 24 years of practice shows how he usually treated a polio patient:

"The muscles which are affected by poliovirus, they are tender, they are painful, so we should give some sort of analgesic medicine, say paracetamol; and of course nutrition, as we mentioned, if the child is malnourished, then we give them. There are other supportive treatments like vitamins B1, B6, and B12 in which we can improve the neural conditions though it is not well documented so far, but we are still using them, giving support to the children. And if the child is having fever, definitely give medicine for the fever or do cold sponging. Cold sponging is the best way to reduce fever. Some medicine will be given if the child is having side-by-side secondary infection problem, then we treat that also. These all are said to be supportive treatment, not specific."

- Private Doctor No.3

On the other hand, other healthcare workers and key informants in our study, including a few young doctors and most auxiliary nurses (ANMs, ASHAs, Aganwadis), lacked adequate knowledge about supportive treatment for polio. Irrespective of literacy, many families had a feeling that they were not given sufficient advice or information for their child. There were existing support systems for paralytic diseases such as financial support by the Government and activities conducted both by the Government and non-governmental organizations (NGOs) where they support the patients by giving out mobility aids and performing orthopedic surgeries, *i.e*. health camps. Yet, only limited individuals even amongst the healthcare providers knew about the existence of these services; needless to say for the families.

Q) "This kind of child who are paralyzed, which are very common in this region, when they come, what kind of suffering they suffer from?"

A) "Mentally and physically, there are both."

Q) "Can you be more specific?"

A) "No, I don't know more about that. But, ah...who are infected with this problem, basically, they are both mentally and physically affected."

- Private doctor No.1

Q) "What kind of care and attention you provide to the child who has polio/are paralyzed?"

A) "Doctor and other people will tell you, I am an ANM and responsible for field work [giving polio drops]. I just refer these kind of child to doctor."

- ASHA No.5

Q) "What kind of treatment and support these kinds of children receive?"

A) "I don't know much about it. Higher official must know about it."

- Male field worker

"We also asked the ANMs for suggestions, but they said that we are sent from there for giving drops and that is what we do."

- Uncle (caregiver) of 2.5 year-old child with polio No.12

Sometimes, the families were given information about these services, but poverty hindered their access. They were unable to fill out forms for applying for financial aid due to illiteracy. Other times, they were not able to afford transportation fees to health camps or rehabilitation centres. There was just one case where the child was provided crutches by the local NGO. Otherwise, parents made crutches from wood and bought tricycle for their children from their own resources.

"They said to write application (for financial support). Now, who has the skills to do all that? What do we know? They had said to write application, but then, who will write..."

- Grandmother of 2 year-old child with polio No.9

"We bought him a cycle for Rs.2500. I used to cry and think when I had to carry him everywhere for nine months and think about his future; why should I wait for the Government; I will buy it from my own money."

- Mother of 5 year-old child with non-polio AFP No.8

Some families expressed no expectations of getting any help because they were poor. They explained that in their experience, poor people never got any help. This made them indifferent in seeking for further help or accessing any means of services regardless of availability.

"See, we are poor people. Poor people never get any help. We don't expect anything from anywhere. Why should we try? How can we try?"

- Grandmother of 3 year-old child with non-polio AFP No. 2

### Basic needs for children with paralysis

The need for adequate medicines for children with AFP was stated by both families and by key informants. Since the government was putting extensive efforts into the polio vaccination campaign in prevention of the disease, some parents expected to receive more support. The demand was for them to act as a 'shield' by offering more help and paying attention to the problems of affected children and their families, regardless of their stool test results.

"Not even money that God looks after, but at least medicines should have been provided. It's a government hospital, at least they should have given medicines."

- Mother of 1.5 year-old child with polio No.1

"If somebody is affected by polio, government should act as a shield. In case people are not capable of managing on their own, then they should be supported by the government."

- Father of 2 year-old child with polio No.5

"Government should help; when government is spending so much money for polio. Gives drops to so many children, going in villages, gives vehicles for it; government should work more rigorously. Something must be done or show them to a good government hospital. Then only one knows whether polio is getting cured or not."

- Father of 3 year-old child with non-polio AFP No.2

"See for them, any paralysis is polio. Ordinary people, why should I make the difference that this is due to poliovirus, and this is not due to poliovirus. My child is affected, my child is affected. I feel that if I have given that dose, that two drops, why should my child get polio? Every child with AFP needed to be suitably compensated as well as rehabilitated, irrespective of polio cases or non-polio cases. That should be the government's responsibility."

- Academic No.4

Education and transportation means were the most desired among the families whose children were left with residual paralysis. Most of the families were labourers, and these children, not being physically productive as the other members of the family, education was the only hope for their future. Their desire was to have their children educated so he or she would 'do something in life', 'not be dependent on anybody', and 'survive'. Transportation means, including mobility aids and transportation arrangements to schools, were demanded from these families. Likewise, many key informants also suggested the importance of education and transportation means for these children and called them their 'basic needs'.

"Education is a must; only education will help him. If he can't do anything but if he will be educated, he may be successful in life."

- *Mother of 2.5 year-old child with polio No.8*

"I just want that by whatever way she is just able to walk on her own. I don't need money. I just want that she should be able to walk on her own."

- Father of 3 year-old child with polio No.16

"Need is to give calibres and make them learn some kind of skills so they can survive in life in spite of being handicapped. Give them vocational training for job oriented profession."

- Private Doctor No.4

"At least some kind of arrangement should be in form of rickshaw or cycle, so that child could reach school."

- Community leader No.3

"People expect everything from the government like money, house, and so on. But if a child is affected with AFP, the most important responsibility of the parents is to provide education to the child because even if the child has paralysis and the child is educated then the child can do some other work just by sitting. The basic need for the child is education. When you give something to people they have the tendency to ask for more, but if they are educated they will be self-dependent."

- International organization No.1

## Discussion

This small carefully conducted qualitative study reveals parent concerns about the extent of care and support available for children diagnosed with AFP. Although particular needs and perceptions varied from family to family, overall the majority claimed to receive very little support from existing government healthcare facilities. Sometimes the reports of family members were contrary to health provider views about care and support. In some cases, we found the provider perspective helpful in understanding the experiences of families. Interviewing various key informants from the community other than healthcare providers enriched our findings by providing a community perspective on polio management. Careful attention was paid towards translation by using expert translator and double-checking transcripts to avoid any misinterpretation. Some of our findings supported those of previous studies investigating healthcare for polio in India [15,16], but as far as we are aware our study is the first to investigate healthcare for children not only with polio but with AFP in India.

Families in our study preferred to visit private-practice doctors. This might have included traditional healers who do not possess a professional medical degree, as we were not able to distinguish the two from information given by the families. This preference for private over government hospitals can also be seen in a house-to-house survey conducted by Varghese in 1990 where it looked at type and place of treatment for children with polio [15]. In the study, only 8% of the paralyzed children were taken to PHCs for treatment, others were taken to private or traditional healers [15].

Interestingly, we found more healthcare providers declared visiting children with AFP for follow up than could be anticipated from the families' interviews, which indicates that potentially some cases were not followed up. This might be due to accessibility of patient homes, difficulty in reaching the moving population, and differences in individual interest for the patients. Many families in this study were unaware of kinds of medical care available for polio. This could be related to low education level of the families, limited access to physiotherapy services in peripheral areas, and financial constraints. Or as we also found in our study, lack of awareness among healthcare providers could have contributed to this poor availability of medical care for the children. Nonetheless, from what we observed from the study area, more fundamental reasons to this gap relate to the poor quality of the public health provision in the area and the function of the AFP surveillance.

Uttar Pradesh is still experiencing its early transition stage in terms of health system [17,18]. Ramani *et al. *have pointed out public health infrastructure is far from satisfactory in poorer states in India as the availability of services is constrained by '(1) non-availability of staff, (2) weak referral system, (3) recurrent funding shortfalls, (4) lack of accountability for quality of care, and (5) poor logistics management of supply of medicines and drugs' [17]. Access to services is an equally important determinant factor in meeting the healthcare needs of people especially in rural areas. However, this need is not being met in areas having minimal or no public transport between PHCs or community health centres and the district referral hospitals as also explained by our respondents [17]. Many families mentioned how the 'poor' do not benefit from any services. This was a particular concern raised by Peters that the rich-favouring distribution of public resources is exercised in Uttar Pradesh [19]. Due to these factors, health indicators of the state are below average in comparison to other states in India [9].

Poor service provision and quality of medical care in Uttar Pradesh compound the problem of identifying children with AFP. For the AFP surveillance has no function in the treatment, care, and support for children affected with AFP or other childhood paralysis, families do not have an appropriate place to seek for further treatment and care. This results in an unbalanced provision of healthcare: on one hand paralyzed children being diagnosed by the AFP surveillance system, and on the other hand, they are left without any treatment or care. Diagnosis followed by adequate treatment and care is a basic right for patients enshrined in the Declaration of Lisbon on the Rights of the Patient [20].

Part of the problem with utilizing the available services might lie in parents' perceptions of the disease and its management. We found that many families did not understand, or some did not want to admit, that polio was an untreatable disease, which made some families constantly seek care from different providers. Healthcare providers we interviewed shared this view, and struggled to provide families with adequate explanations about treatment and care. Difficulty in accepting the permanence of the disease increased family members' resentment against immunisation, which they are told prevents the disease. Despite multiple doses of OPV, the occurrence of polio is of concern, and this issue is debated elsewhere [21,22]. It is interesting to note that no feeling of guilt for their children was heard from our respondents, while when Kishore explored the same issue back in 2003, it was present in all ten parents with children with polio [16]. This change in the attitude of the families is possibly due to the growing resentment against immunisation over time.

No definite diagnosis was given for those whose stool exam was negative for polio. Two of the nine children with non-polio AFP were left with residual paralysis, which indicates the possibility of polio despite their stool status. There could be a possibility of false negative results of polio from stool samples, as there are to any other diagnostic tests. Furthermore, OPV includes live attenuated poliovirus which sometimes cause vaccine-associated paralytic poliomyelitis (VAPP). Since this OPV is administered to children every month in certain areas, there is a chance that some of these cases with residual paralysis could be the VAPPs [21,23]. For the parents, it did not matter whether or not their children's final diagnosis was polio or not. For them, all paralysis was taken as polio, and what all mattered to them was to have their children treated and recover from paralysis.

Arora *et al. *stated that we have to pay more respect to the voices of the marginalized population and the most peripheral health workers, not to forget them under the robustness of international advocacy, in order to succeed in polio eradication [24]. This also stands true when dealing with children with AFP, as they are part of the programme. From the voices heard from our study, healthcare for children with AFP needs to be improved. Healthcare providers and families of children with AFP need adequate information on available treatment and services, and action is needed to improve knowledge and awareness. In addition, integration of AFP surveillance with other services that provide services for people with disabilities is essential.

After an outbreak of polio in Tajikistan in 2010 with 712 cases of AFP, the Ministry of Health of Tajikistan and the WHO regional office built up their rehabilitation capacity by hiring a rehabilitation consultant, arranging training courses for local doctors, and visiting homes of affected children to give parents training in physiotherapy [25]. To continue regular training for medical personnel and parents, a stakeholders' meeting was held for partner organizations and societies for disabled people in the country [25]. By utilizing the system and network established for the Pulse Polio Programme, a similar approach would be possible in India. Integration of AFP surveillance into the already-existing scheme of Community Based Rehabilitation (CBR) could be another approach. CBR programmes provide community-based medical, rehabilitative, educational, social work services to disabled people [26]. Although CBR is also in the process of development [27], the two services could collaborate with each other; with AFP surveillance enabling early identification of paralytic cases and CBR programmes acting as the caregiver.

The gains made with these approaches are likely to be minimal if the quality of the public health system does not improve, as there are broader, underlying problems. To tackle this, measures are needed to modify the whole public health system: its infrastructure, function, and performance in these poorer states of India.

## Conclusions

Despite the high number of children diagnosed with AFP as part of the global polio eradication programme, they are not provided with sufficient medical care following their diagnosis in Uttar Pradesh, India. Measures should be taken to improve their quality of life, especially those with residual paralysis.

## Competing interests

The authors declare that they have no competing interests.

## Authors' contributions

RRY contributed in the design, data collection, and qualitative analysis of the study as well as the draft of the manuscript. KA and HS provided input into the design of the study and into the content and presentation of the final manuscript. AD contributed in the design and supervised fieldwork. All authors read and approved the final manuscript.

## Pre-publication history

The pre-publication history for this paper can be accessed here:

http://www.biomedcentral.com/1471-2458/12/229/prepub

## Supplementary Material

Additional file 1 **Table S1: Topic guidelines for interviews with the parents with AFP**. **Table S2**: Topic guidelines for interviews with healthcare workers and key informants. **Table S3a**: Expanded data for characteristics of confirmed polio cases in the study. **b**: Expanded data for non-polio AFP cases in the study. **Figure S1**: Map of Muzaffarnagar and cases of polio. Click here for file
