# Supplementary material for: Support for children identified with acute flaccid paralysis under the global polio eradication programme in Uttar Pradesh, India: a qualitative study
Source: BMC Public Health. 2012 Mar 22;12:229. doi: 10.1186/1471-2458-12-229 (PMC3331818; doi:10.1186/1471-2458-12-229)

Table S1: Topic guidelines for interviews with the parents with AFP

Topic 1. Experience around the Usage of Health Facilities at the Time of Onset of the Disease (Patient History)

1.1 What were the symptoms of your child when you first noticed something was wrong?

[Probe: How did you first notice something was wrong with your child? How did you come to know about your child's disease?]

1.2 When was it?

1.3 Where did you take him/her to? What doctor?

1.4 Why did you take her to [the place mentioned in 1.3]?

1.5 What happened after that?

[Probe: What kind of exams did you have to go through? What kind of treatment did you get? Did you have to go somewhere else? etc.]

1.6 What information did doctors give you for your child's disease?

1.7 Did your child do a stool test?

1.8 Where did your child have the stool test done? At hospital or somebody come to your house?

1.9 What kind of treatment, support, or care did you get after the stool test from the people visiting you at your house [ANMs, doctors]?

1.10 What information did they provide you with polio about the illness [regarding the characteristic of the disease, e.g., prognosis]?

1.11 Also, what information did they provide you regarding treatment, support, and care of the disease [polio]?

1.12 What happened *when* the result of the stool test came?

[Did some one explain the result to you? If yes, what kind of explanation did you get? If no, why didn't you get the result?]

1.13 What happened *after* you got the result for the stool test?

[Were you still visited by the doctors and ANMs?]

1.14 Were you visited after 2 months time after you went to hospital for the first time? (60-day follow-up to see residual paralysis)

1.15 What do you think of these services by the government they've got for your child?

Topic 2. Treatment, support & care

2.1 Where are you getting treatment/support/care for your child? If not getting any, then why?

2.2 What kind of treatment/support/care?

2.3 Why did you choose to get the service from [answer of 2.1]?

2.4 What were your difficulties getting treatment, support, and care at the time of onset of polio?

[e.g., cost, transportation, etc.]

2.5 What are your difficulties now getting treatment, support, and care for your child?

2.6 What did/do you feel about the cost of treatment?

2.7 How is the treatment cost affecting your family?

Topic 3. Difficulties with the symptom of the child

3.1 How is your child now?

[Probe: recovered, symptoms still remaining (e.g., cannot sit up, cannot walk)?]

3.2 What are your challenges and difficulties of looking after this child? Please share actual experiences.

Topic 4. Needs and suggestions for the future

4.1 What kind of information should have been helpful for you at the time when you were visiting hospitals? Also for now and for the future?

4.2 What kind of help (support and care) did you needed or think situation would have been better if you had?

4.3 What are your suggestions for support and care for a child like yours?

4.4 Any other suggestions?

Topic 5. Experience and Understanding of Polio and Polio Vaccine

5.1 How many times your children has gone through this drop?

5.2 Did you ask the doctor why your child got polio despite you giving the drops? What did they tell you?

5.3 In what way have your views of the polio drops changed after your child having the disease?

Topic 6. Experience with the Routine Immunisation

6.1 Have you been going through immunisation other than polio? If yes, what? If no, why not?

6.2 What do you think are the differences between the polio drops and other immunisation?

[Probe: access is difficult, cost, lack of information, etc.]

Topic 7. Experience and Perceptions around Child Health and Healthcare

7.1 Please explain your daily meal. (How many times? What do you eat?)

7.2 Do your child get sick often? If yes, what kind of disease?

7.3 What kind of treatment or support / care do they get for this? From where?

7.4 Do you see any differences between the support you are getting for paralysis and for other diseases? What are your thoughts around this?

7.5 What kind of services do you need for your child?

7.6 How do you think health services for children in your area can be improved?

Table S2: Topic guidelines for interviews with healthcare workers and key informants

*Questions are excluded if not relevant to the respondent.

Topic 1. Perceptions around Child Health and Healthcare

1.1 How is the health condition of children in the area?

1.2 What kind of disease is commonly seen among children?

1.3 What do you think are the priorities taken by the community regarding child health?

1.4 What do you think of the health system in the area? How is it affecting child health?

1.5 What are the things that you think should be done to improve the health condition of children in your area?

Topic 2. Perceptions and Experiences with Vaccinating Children

2.1 What is your perception on the polio drops?

2.2 Do people come to your clinic for polio drops?

2.3 What information do you give to mothers when you are giving the children the polio drops?

2.4 Do you face any resistance from mothers? If yes, who resist and for what reasons?

2.5 Have you seen any changes in mothers' view/perception to the polio drops over the years?

2.6 What impact do you think the polio drops are having on the community?

2.7 How is routine immunisation in the area? If it is not good, what are the reasons behind?

Topic 3.Perceptions and Experiences with the AFP Cases including Poliomyelitis

3.1 How is a child diagnosed as acute flaccid paralysis (AFP)?

3.2 What happens to a child after a diagnosis is given as acute flaccid paralysis?

3.3 Do you know any child who is diagnosed as acute flaccid paralysis? If yes, is there a trend to what kind of family they come from?

3.4 What kind of treatment or care are the children with acute flaccid paralysis getting? (Are they getting followed-up? Are they getting any treatment or care/support? Do you visit them?)

3.5 Do you know any polio cases in the community? If yes, is there a trend to what kind of family they come from?

3.6 What kind of treatment or care are the children with polio getting? (Are they getting followed-up? Are they getting any treatment or care/support? Do you visit them?)

3.7 What do you think are the needs of these children?

3.8 What do you think should be done for the children with acute flaccid paralysis?

3.9 What do you think should be done for the children with polio?

3.10 What do you think of the AFP surveillance system? (e.g., process, effectiveness, quality, etc.)

Table S3a: Expanded data for characteristics of confirmed polio cases in the study

	Case Number								
	1	2	3	4	5	6	7	8	9
Gender	F	F	M	F	M	F	M	M	M
Age at interview	1.5 yr.	3.5 yr.	3 yr.	2 yr.	2 yr.	4 yr.	2 yr.	2.5 yr.	2 yr.
Age at onset	1 yr.	2.5 yr.	2 yr.	11 m.	1 yr.	2.5 yr.	1.5 yr.	1.3 yr.	1.5 yr.
Type of poliovirus*	P3	P3	P3	P3	P3	P3	P3	P1	P1
Stool exam result*	P3 Wild/P3 Wild	P3 Wild/P3 Wild	P3 Wild/P3 Wild	P3 Wild/P3/Wild	P3 Wild/NPEV	P3 Wild/P3 Wild	P3 Vaccine/P3 Wild	P1 Wild/P1 Wild	P1 Wild/P1 Wild
No. of OPV through SIAs*	6	14	12	6	0	14	13	10	8
No. of OPV through RI*	2	0	1	1	6	1	0	0	0
OPV doses received before illness*	8	14	13	7	6	15	13	10	8
Residual paralysis	No	No	No	No	No	No	No	Yes	Yes
Religion	Muslim	Muslim	Muslim	Muslim	Muslim	Muslim	Muslim	Muslim	Muslim
Occupation of parents	labourer	labourer	juice vendor	labourer	labourer	labourer	labourer	labourer	labourer
Literacy of parents	illiterate	illiterate	literate	literate	literate	illiterate	illiterate	illiterate	illiterate

	Case Number							
	10	11	12	13	14	15	16	17
Gender	F	M	M	M	M	M	F	M
Age at interview	6.5 yr.	2 yr.	2.5 yr.	2 yr.	2.5 yr.	2.5 yr.	3 yr.	9 yr.
Age at onset	6 yr.	10 m.	1.5 yr.	6 m.	1.8 yr.	1.7 yr.	2 yr.	8 yr.
Type of poliovirus*	P3	P3	P1	P1	P1	P3	P1	P3
Stool exam result*			P1 Wild/P1 Wild	P1 Wild/Negative	P1 Wild/P1 Wild	P3 Wild/P3 Wild	P1 Wild/NPEV	P3 Wild/P3 Wild
No. of OPV through SIAs*			6-8	16+	9	14	14	15
No. of OPV through RI*	NR	NR	0	4	0	0	0	0
OPV doses received before illness*			6-8	20+	9	14	14	15
Residual paralysis	No	No	Yes	Yes	No	No	Yes	No
Religion	Muslim	Muslim	Hindu	Hindu	Muslim	Muslim	Muslim	Muslim
Occupation of parents	labourer	labourer	labourer	labourer	shop owner	labourer	businessman	rickshaw puller
Literacy of parents	illiterate	illiterate	illiterate	literate	literate	illiterate	literate	illiterate

NR: not recorded; SIAs: Supplementary Immunisation Activities;RI: Routine Immunisation; NPEV: non-polio enterovirus

Table S3b: Expanded data for non-polio AFP cases in the study

	Case Number								
	1	2	3	4	5	6	7	8	9
Gender	F	M	M	M	M	F	M	M	M
Age at interview	2 yr.	3 yr.	7 yr.	4 yr.	8 m.	4.5 yr.	3 yr.	5 yr.	7 yr.
Age at onset	1.5 yr.	1.9 yr.	6.3 yr.	3.5 yr.	5 m.	4 yr.	2 yr.	4.3 yr.	6 yr.
Stool exam result*	Negative/Negative	NPEV/NPEV	Negative/Negative	Negative/Negative	Negative/Negative	Negative/Negative	P3 Vaccine/P3 Vaccine	Negative/Negative	Negative/Negative
No. of OPV through SIAs*	10	12	25	20	NR	20	12	24	20
No. of OPV through RI*	2	3	0	1		0	3	1	0
OPV doses received before illness*	12	15	25	21		20	15	25	20
Residual paralysis	No	Yes	No	No	No	No	No	Yes	No
Religion	Muslim	Muslim	Muslim	Muslim	Muslim	Muslim	Hindu	Hindu	Muslim
Occupation of parents	furniture shop	tailor	driver	labourer	labourer	labourer	sugar mill employee	labourer	labourer
Literacy of parents	literate	illiterate	illiterate	literate	illiterate	illiterate	literate	illiterate	illiterate

NR: not recorded; SIAs: Supplementary Immunisation Activities;RI: Routine Immunisation; NPEV: non-polio enterovirus

* Data retrieved from medical record

Figure S1: Map of Muzaffarnagar and cases of polio

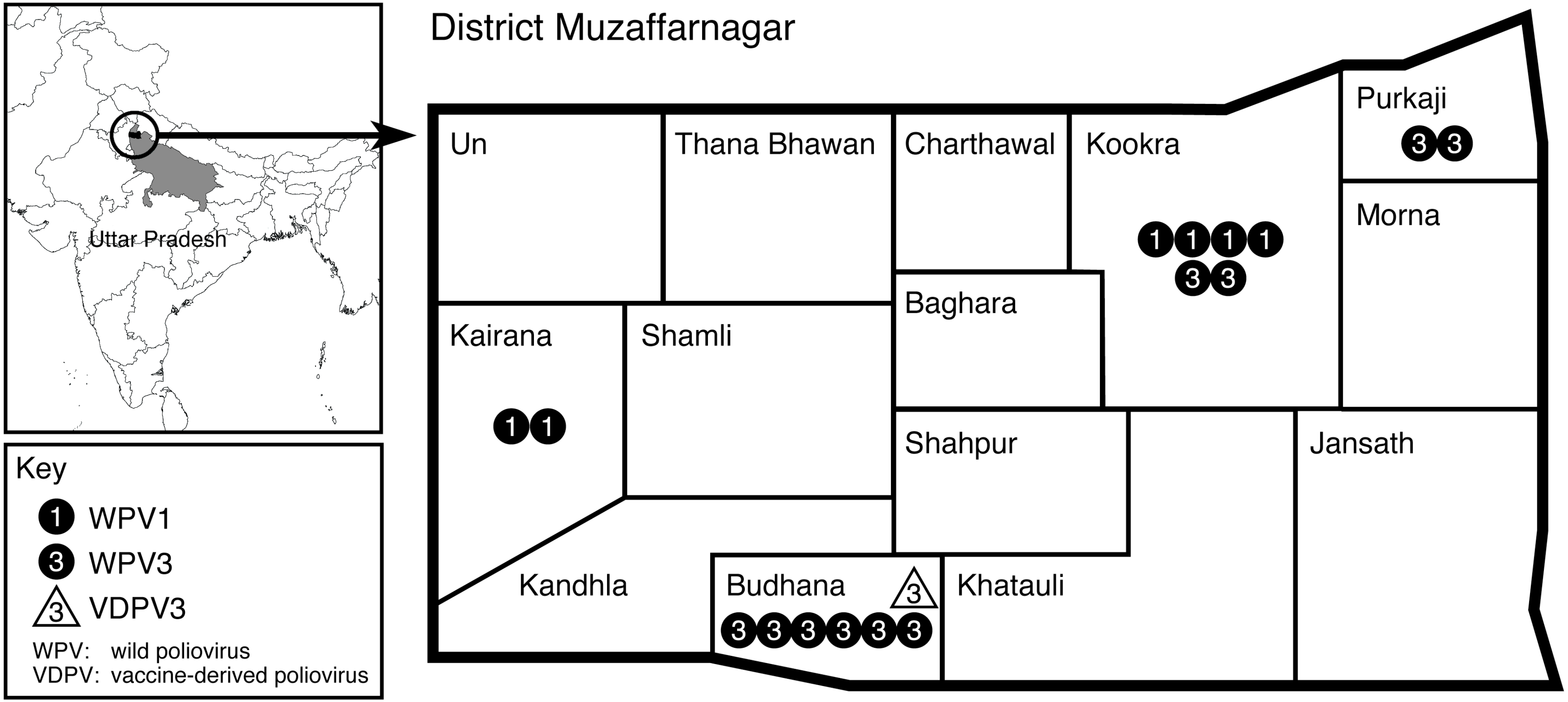

Supplement: Additional file 1 — Table S1: Topic guidelines for interviews with the parents with AFP. Table S2: Topic guidelines for interviews with healthcare workers and key informants. Table S3a: Expanded data for characteristics of confirmed polio cases in the study. b: Expanded data for non-polio AFP cases in the study. Figure S1: Map of Muzaffarnagar and cases of polio. [file 1471-2458-12-229-S1.PDF]
